# Genetic Enhancement of Memory and Long-Term Potentiation but Not CA1 Long-Term Depression in NR2B Transgenic Rats

**DOI:** 10.1371/journal.pone.0007486

**Published:** 2009-10-19

**Authors:** Deheng Wang, Zhenzhong Cui, Qingwen Zeng, Hui Kuang, L. Phillip Wang, Joe Z. Tsien, Xiaohua Cao

**Affiliations:** 1 Shanghai Institute of Brain Functional Genomics, the Key Laboratories of MOE and STCSM and College of Life Sciences, East China Normal University, Shanghai, China; 2 Brain and Behavior Discovery Institute and Department of Neurology, School of Medicine, Medical College of Georgia, Augusta, Georgia, United States of America; 3 Yunnan Banna Primate Model Research Center, Xishuang-Banna, Yunnan, China; L'université Pierre et Marie Curie, France

## Abstract

One major theory in learning and memory posits that the NR2B gene is a universal genetic factor that acts as rate-limiting molecule in controlling the optimal NMDA receptor's coincidence-detection property and subsequent learning and memory function across multiple animal species. If so, can memory function be enhanced via transgenic overexpression of NR2B in another species other than the previously reported mouse species? To examine these crucial issues, we generated transgenic rats in which NR2B is overexpressed in the cortex and hippocampus and investigated the role of NR2B gene in NMDA receptor-mediated synaptic plasticity and memory functions by combining electrophysiological technique with behavioral measurements. We found that overexpression of the NR2B subunit had no effect on CA1-LTD, but rather resulted in enhanced CA1-LTP and improved memory performances in novel object recognition test, spatial water maze, and delayed-to-nonmatch working memory test. Our slices recordings using NR2A- and NR2B-selective antagonists further demonstrate that the larger LTP in transgenic hippocampal slices was due to contribution from the increased NR2B-containing NMDARs. Therefore, our genetic experiments suggest that NR2B at CA1 synapses is not designated as a rate-limiting factor for the induction of long-term synaptic depression, but rather plays a crucial role in initiating the synaptic potentiation. Moreover, our studies provide strong evidence that the NR2B subunit represents a universal rate-limiting molecule for gating NMDA receptor's optimal coincidence-detection property and for enhancing memory function in adulthood across multiple mammalian species.

## Introduction

The NMDA receptor, the molecular switch for synaptic plasticity [1], [2], [3], [4] and learning and memory [5], [6], [7], [8], is composed of NR1 and at least of one of the NR2 (A, B, C, and D), or NR3 (A and B) subunits [9], [10]. The NR2A and NR2B subunits are predominant subunits in excitatory pyramidal cells of the cortex and hippocampus to form the receptor complex with the NR1 subunit and to underlie the receptor's coincidence-detection function [11]. Over the time course of postnatal development there is a dynamic change in the ratio of NR2A over NR2B, with increased production of NR2A-containing NMDARs and reduced NR2B-containing NMDARs as the postnatal brain develops into the adult stage [11], [12], [13], [14]. This change in the subunit composition has been postulated to be crucial for underlying the gradual shortening of NMDA currents and increased threshold for synaptic plasticity induction [7], [15]. A pharmacological experiment using antagonists selectively blocking NR2A- or NR2B-containing NMDARs reported that the NR2A-containing NMDARs was designated for the induction of LTP, whereas NR2B-containing NMDARs was exclusively responsible for the induction of LTD in the CA1 region of the rat hippocampus [16]. However, other studies in rat CA1 region using the similar pharmacological methods reported otherwise [17], [18], [19]. One alternative way to examine the validity of this hypothesis is to overexpress the NR2B subunit or NR2A subunit and examine its effects on LTP and LTD in the rat hippocampus. Since the NR2A subunit is known to be much more predominant in the adult hippocampus than that of the NR2B subunit in the adult brain, overexpression of the rate-limiting NR2B subunit can represent a simple and yet crucial test for the “NR2A-for-LTP/NR2B-for-LTD” hypothesis. Due to the fact that the majority of those studies were done in rats, we purposely generated the transgenic rats, instead of mice, and overexpressed the NR2B subunit in the forebrain regions such as the hippocampus and cortex. This transgenic rat system has allowed us to test an important prediction of the “NR2A-for-LTP and NR2B-for-LTD” hypothesis: if NR2B-containing NMDARs were specialized in triggering LTD, overexpression of NR2B should selectively affect CA1-LTD (producing larger LTD), and has no or only a small effect on CA1-LTP. In addition, the NR2B rats would also permit us to examine whether they possess superior learning and memory functions in comparison to their wild-type littermates.

## Results

### Production and biochemical characterization of NR2B Transgenic rats

**Figure 1:**
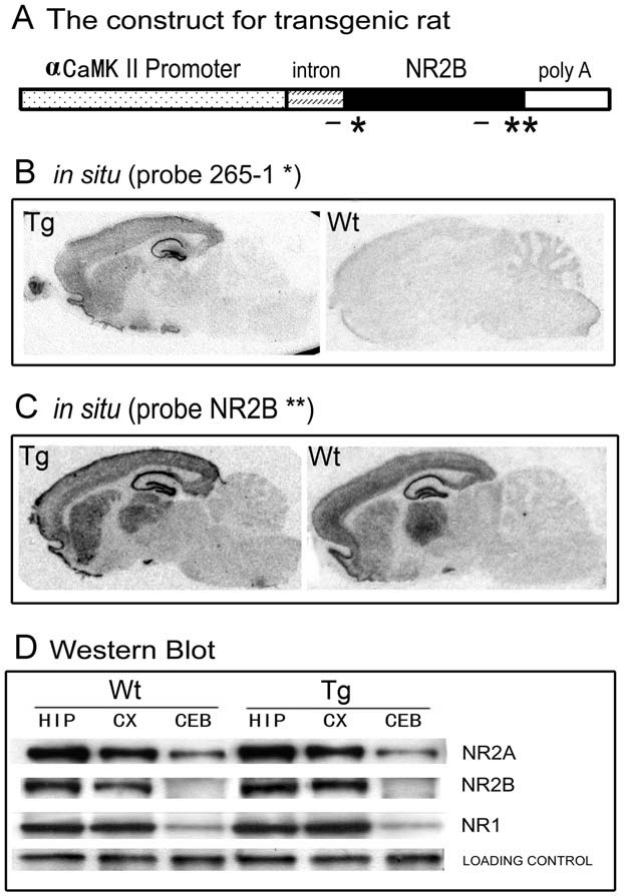
Production and Basic Characterizations of NR2B Transgenic Rats. A: The construct for making NR2B transgenic rats. An 8.5 kb alpha-CaMKII promoter was used to drive NR2B overexpression. The small solid bar indicates the probes used for *in situ* hybridization, (*) indicates the probe for vector of 265-1 intron, (**) represents the probe within the NR2B encoding sequence. B: *in situ* hybridization using transgene-specific probe of 265-1. The transgene mRNA expression showed forebrain-specific expression pattern. No signal was detected in the wild-type brain. C: *in situ* hybridization using a probe detecting NR2B mRNA expression. D: Western blots measure the amount of NR2A, NR2B and NR1 expression in the rat brains. Hippocampus: HIP, Cortex: CX, Cerebellum: CB.

The forebrain-specific CaMKII promoter was used to drive NR2B transgene expression in the rat hippocampus and cortex (Figure 1A), and we injected the linearized transgene construct into the fertilized embryos of Long Evans female rats. Despite a certain technical variation associated with microinjection procedures in this rat strain, we were able to produce a transgenic line with high expression of the NR2B construct. Our *in situ* hybridization confirmed the expression of mRNA of the NR2B transgene in the forebrain (Figure 1B and C). The western blots also show increased amount of NR2B proteins in the synaptosome preparation (Figure 1D). The offspring of this line grow normally (Supporting Figure S1) and exhibit indistinguishable behavior such as locomotion and exploratory behavior (data not shown).

### Enhanced Recognition Memory in NR2B Transgenic Rats

Since overexpression of NR2B is reportedly capable of enhancing memory function in mice [7], [20], [21], we wonder whether overexpression of NR2B would also benefit memory function in rats. Thus, we conducted a set of behavioral experiments, namely novel object recognition test, hidden-platform water maze, and T-maze spatial working memory tests, to see if the transgenic rats exhibit better memory performances.

**Figure 2:**
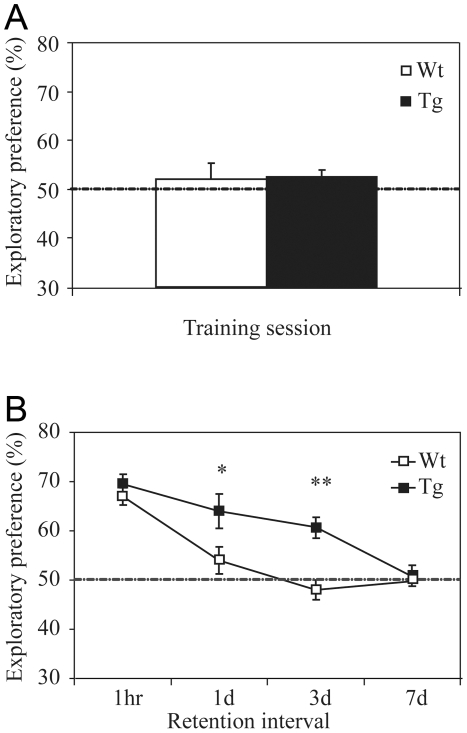
Enhancement of Novel Object Recognition Memory in Transgenic Rats. A: Similar exploratory preference between transgenic and wild-type rats during training session of the novel object recognition tests. B: Enhanced exploratory preference in transgenic rats in 1-day and 3-day retention tests.

In the novel object recognition test, we found that there was no significant difference in the amount of time spent on exploring the two objects during the training session, as shown by the exploratory preference (Figure 2A). This indicates both the transgenic rats and their wild-type littermates have the same levels of motivation and curiosity for exploring these two objects. Furthermore, we found that both transgenic and wild-type rats exhibited the similar levels of preference toward the novel object at the 1 hour retention test (Figure 2B). Interestingly, when retention tests were conducted at longer duration, such as 1 day and 3 days later, the transgenic rats exhibited much stronger preference for the novel object than wild-type rats (Figure 2B). A post hoc analysis by using Dunnett's test reveals a significant difference between wild-type and transgenic rats at the 1-day (F_(1, 26)_ = 5.25, p<0.05) and 3-day retention tests (F_(1, 19)_ = 19.77, p<0.01). However, when the retention duration was further increased to 1 week, the transgenic rats no longer exhibited any preference (Figure 2B), indicating both types of rats have forgotten the objects they explored initially. These experiments suggest that the transgenic rats outperformed control rats in this recognition memory test.

### Enhanced Spatial Reference Memory in NR2B Transgenic Rats

**Figure 3:**
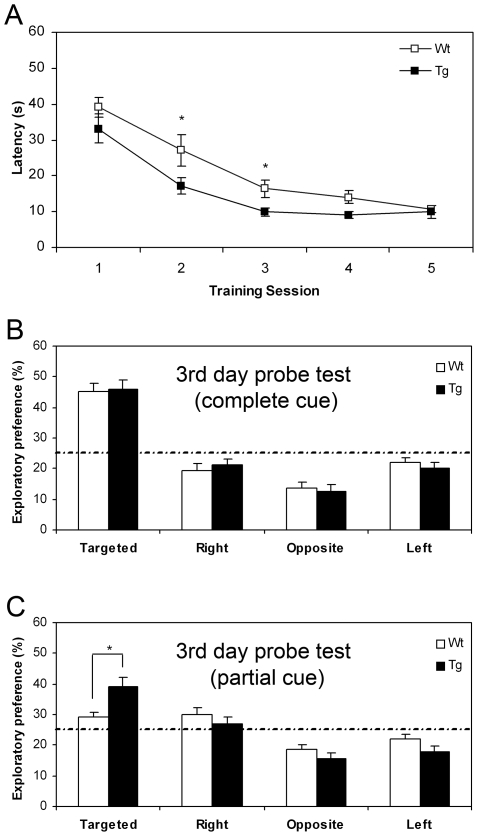
Enhanced Performance in the Hidden-platform Water Maze Task by NR2B Transgenic Rats. A: Escape latency in water maze training. Transgenic rats spent the significant less time to find the platform during the second and third training sessions. B: At the end of the third training session, spatial memory function was tested under full cue condition. Transgenic and Wt rats exhibited equal amount of strong preference for the target quadrant under full-cue conditions (Student's t-test, *P<0.05). C: Place preference was further tested at the end of the third training session under the partial cue condition. Transgenic rats spent more time in the target quadrant than other quadrants, in comparison to that of wild-type rats.

We then measured the spatial memory function using the hidden-platform water maze. We first assessed the escape latency during the water maze training sessions. We found that transgenic rats spent the significant less time to find the platform during the second and third training sessions (Figure 3A). At the end of the third training session, we examined the spatial memory function by measuring the place preference under the full cue condition. Transgenic and wild-type rats exhibited equal amount of strong preference for the target quadrant under full-cue conditions (Figure 3B). Interestingly, when we increased the task difficulty by using the partial cue condition, we found that transgenic rats outperformed their wild-type counterparts. They spent significantly more time in the target quadrant than other quadrants, in comparison to that of wild-type rats (Figure 3C), suggesting that the transgenic rats were better in this spatial memory test. It is worthy to note that at the end of 5^th^ training session, there is no longer any difference in place preference under either full cue or partial cue conditions (Supporting Figure S2).

### Enhanced Spatial Working Memory in NR2B Transgenic Rats

**Figure 4:**
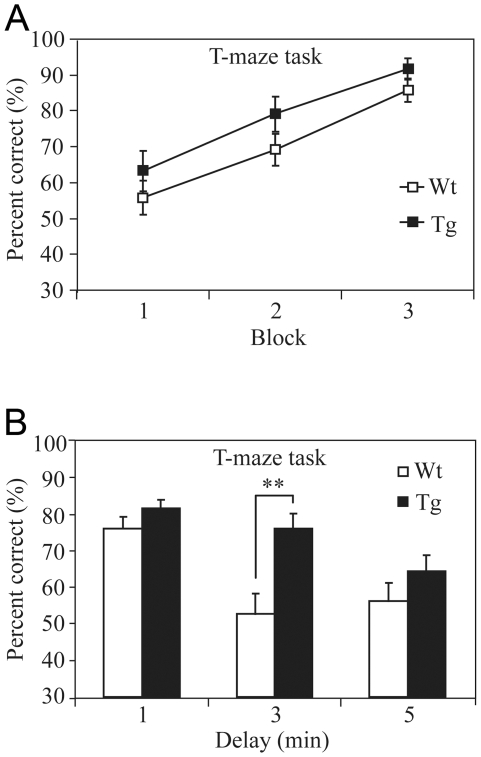
Enhancement of Spatial Working Memory in Transgenic Rats. A: NR2B transgenic rats exhibited the similar learning curve in comparison to that of controls during the T-maze spatial working memory test. The delay interval between the sample-run and trial-run is 15 seconds. B: Spatial working memories were tested using various intervals. Better performance in the transgenic rats was noted in the 3-min delay non-matching to place test. Data are expressed as mean±SEM. Asterisk, p<0.05; double asterisk, p<0.01; post hoc analysis.

We also tested the working memory function in NR2B transgenic rats by using the elevated T-maze non-matching-to-place task [22]. Each individual trial of this task is consisted of a sample-run and a choice-run. On the sample-run, the rats were forced to run down either the left or right arm (in randomized sequence) to obtain a food reward. On the choice-run which starts 15 seconds after the sample run, the rats were placed at the end of the start arm and allowed a free choice of either goal arm (the correct arm should be non-matching to the sample-run arm). The NR2B transgenic rats exhibited the similar learning curve in comparison to that of the controls (Figure 4A). Upon the completion of the training trials, we increased the difficulty of the tasks by systematically varying the time interval between the sample- and the choice-run (ranging from 1 minute to 3- or 5-minutes). Interestingly, NR2B transgenic rats exhibited superior performances in comparison to those of their control littermates when the interval between sample-run and test-run was delayed 3 minutes (Figure 4B) (F_(1, 25)_ = 10.97, p<0.01). For the 5-minute-delay interval, although there seems to be a tendency that Tg rats performed better than the wild-type littermates, there was no statistically significant difference.

### Enhanced LTP of Hippocampus in Transgenic Rats

**Figure 5:**
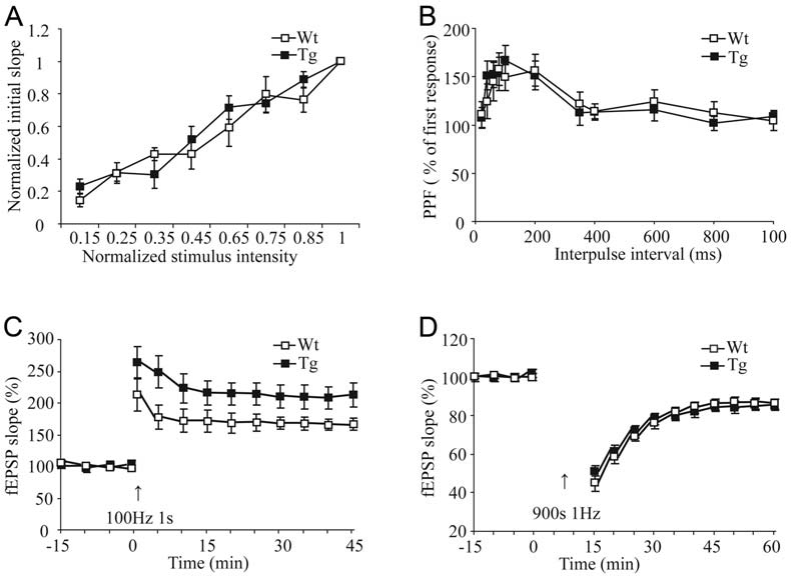
Synaptic Transmission and Plasticity in the Transgenic Rat Hippocampus. A: Normal CA3-CA1 input/output curve in Tg hippocampal slices. B: Normal paired-pulse facilitation at CA3-CA1 in Tg hippocampal slice. C: Tetanic stimulation induced larger LTP in transgenic slices than that of control. D: Low-frequency stimulation evoked the similar LTD between Tg and WT slices. Data were presented as the mean±SEM. Student's *t*-test was used for statistical analysis.

To investigate the effects of NR2B overexpression on the hippocampal synaptic transmission and plasticity in the transgenic NR2B rats, we used slice recording techniques and measured synaptic properties in the Schaffer collateral-CA1 path of adult rats (2–3 months old). We found that there was no significant difference in basal synaptic transmission (Figure 5A. WT, n = 6 slices/3 rats; Tg, n = 8 slices/4 rats) and paired-pulse facilitation (Figure 5B. WT, n = 9 slices/3 rats; Tg, n = 14 slices/5 rats) between transgenic and wild-type hippocampal slices. This suggests that the overexpression of the NR2B transgene did not change presynaptic function and basic synaptic transmission. However, we found that a single tetanic stimulus evoked significantly larger long-term potentiation (LTP) in transgenic slices than that in the wild-type slices (Figure 5C; Tg, n = 9 slices/7 rats, 209±16.82%; WT, n = 9 slices/6 rats, 166±8.67%; p<0.05 in comparison to transgenic rats). This form of enhanced LTP was NMDA receptor-dependent because application of 100 µM AP-5 in the perfusion solution completely blocked LTP in transgenic slices (data not shown).

We next investigated long-term depotentiation (LTD) by using the 1-Hz low-stimulation frequency protocol. No significant difference in LTD was observed between wild-type (Figure 5D, 86±2.42%; n = 12 slices/5 rats) and transgenic slices (85±2.56%; n = 12 slices/4 rats, t-test, p = 0.869 vs. wild-type).

### Involvement of NR2A Subunits in LTP in Transgenic and Wt Rats

**Figure 6:**
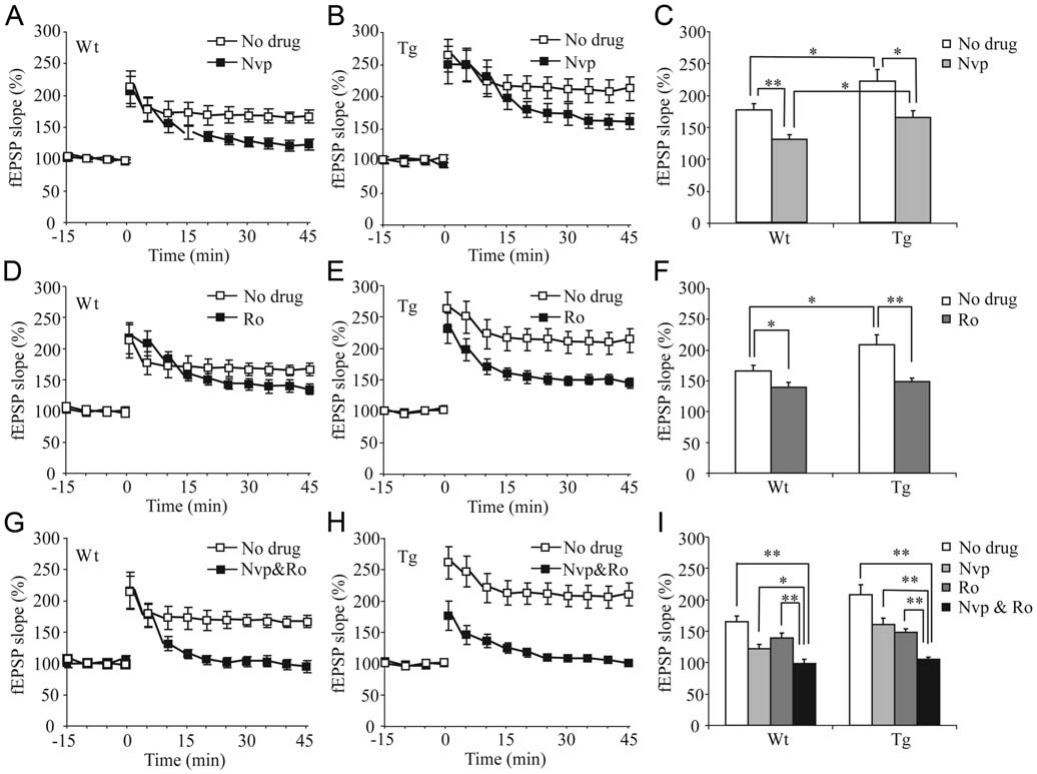
The Role of NR2B Subunit in Enhanced CA1-LTP in Transgenic Hippomcampal Slices. A: Effects of NR2A-selective antagonist, NVP-AAM077, on wild-type slices. B: NVP-AAM077 also significantly reduced, but not completely blocked, CA1 LTP in transgenic slices. C: Statistical analysis shows the effects of NVP-AAM077 on LTP in both Tg and Wt slice. It indicates a significant involvement of NR2A-containing NMDARs in CA1-LTP induction in both Tg and WT slices. D: There was a significant, but small, effect of NR2B-selective antagonist, Ro25-6981, on CA1-LTP in wild-type slices. E: Ro25-6981 had much larger effect on CA1-LTP in transgenic slices, but it did not completely block LTP in transgenic slices. F: Summarized effects of Ro25-6981 on CA1-LTP in both Tg and Wt slice. G: Combination of NVP-AAM077 and Ro25-6981 completely blocked the formation of CA3-CA1 LTP completely in the wild-type slices. H: Complete blockage of CA1-LTP in transgenic slices by combined application of NVP-AAM077 and Ro25-6981. I: Statistical analysis shows the combined effects of NVP-AAM077 and Ro25-6981 on CA1-LTP. (Data were presented as the mean±SEM. Student's *t*-test was used for statistical analysis).

To assess the relative contribution of the NR2A and NR2B subunits to LTP, we added pharmacological antagonists to our hippocampal preparations. We first used NR2A selective antagonist, NVP-AAM077. We found that LTP in wild-type slices was significantly reduced but not completely blocked by 0.4 µM NVP-AAM077 (Figure 6A, no drug, n = 9 slices/6 wt rats, 166±8.67%; with drug, 122±7.64%; n = 6 slices/5 rats, t-test, p<0.05). Similarly, NVP-AAM077 also reduced the level of LTP in the transgenic slices (Figure 6B. 209±16.82% without the drug, 161±9.86% with the drug). It is important to note that even after treatment with NVP-AAM077, LTP in Tg slices (161±9.86%, n = 6 slices/5 rats) was still significantly larger than that of WT slices (122±7.64%, n = 7 slices/5 rats; p<0.05) (Figure 6C).

### Requirement of NR2B Subunits for LTP in Transgenic and Wt Rats

We then examined the role of NR2B-containing NMDARs in CA1-LTP using NR2B-selective antagonist, Ro 25-6981. We found that 3 µM Ro 25-6981 mildly reduced LTP on the wild-type slices (Figure 6D, 139±8.58% drug treated; n = 8slices/6 rats, t-test, p<0.05), whereas the same drug treatment greatly decreased LTP in the transgenic slices (Figure 6E, 148±6.38% drug treated; n = 9 slices/5 rats, compared to the untreated 209±16.82%, p<0.01). Importantly, under the Ro25-6981 treatment LTP level in Wt slices was statistically similar to that of Ro25-6981-treated Tg slices (Figure 6F; wild-type, 139±8.59%; n = 8 slices/6 rats; transgenic, 148±6.38%; n = 9 slices/5 rats; p = 0.405).

Finally, we conducted the LTP recording experiments using combined NVP-AAM077 and Ro25-6981 protocols. The combined application of NVP-AAM077 and Ro25-6981 produced additive effects and together these two drugs were able to completely block LTP induction in the wild-type slices (Figure 6G, 99±6.87%; n = 8 slices/3 rats.) as well as in the transgenic slices (Figure 6H, 105±3.53%; n = 7 slices/3 Rats). Statistical analyses show there was no significant difference between the genotypes (Figure 6I, p>0.05).

## Discussion

Using genetic approach, we have examined two major hypotheses: 1) subunit composition of the NMDA receptor controls the direction of synaptic plasticity, specifically, NR2A-containing NMDARs triggers CA1-LTP, whereas NR2B-containing NMDARs triggers CA1-LTD [16]. 2) the NR2B gene is a universal genetic factor that acts as rate-limiting molecule in controlling memory function across multiple animal species. As to the first hypothesis, one crucial prediction of this “NR2A triggering LTP/NR2B triggering LTD” hypothesis would be that overexpression of NR2B in rat hippocampus should lead to more robust LTD and no change in LTP. In contrast to the prediction for enhancement of CA1-LTD, our results demonstrate that overexpression of the NR2B subunit in the rat hippocampus had no observable effect on CA1-LTD, but rather resulted in enhanced CA1-LTP. Again, the selective enhancement of LTP but not LTD is similar to what was previously described in NR2B transgenic mice [7], [21].

More importantly, by making transgenic rats instead of transgenic mice, we can avoid the argument of species difference [23], [24] and make our measurements more directly comparable to the results using rat preparations. It should be noted that our present study focused on NR2B overexpression. It would be useful to embark on similar efforts in examining the effect of NR2A overexpression in near future.

Our detailed analyses using NR2A- and NR2B-selective antagonists reveal that the larger LTP in transgenic hippocampal slices was due to contribution from the increased NR2B-containing NMDARs. Taken together, our experiments clearly demonstrate that NR2B is not designated specifically for triggering LTD, but rather plays a crucial role in controlling the LTP process. This basically nullifies “NR2A triggering LTP/NR2B triggering LTD” hypothesis.

One of the major interests in cognitive neuroscience community is whether it is possible to enhance memory function in the normal brain. Ten years ago, we reported the successful generation of transgenic mice with enhanced learning and memory via enhancing the NMDA receptor's coincidence-detection function [7]. However, one of the major questions followed after our initial work in mice is whether the NR2B gene is a universal molecule that represents the rate-limiting molecular switch for controlling synaptic plasticity and memory function across multiple animal species. Although the extrapolation of the scientific conclusions from one species to another is logical given the overwhelming similarity in the molecular composition, expression distribution, and physiological properties of the NMDA receptors ranging from mice to rats and from monkeys to humans, it should not serve as a complete substitute for actual experiments. Thus, it is crucial to perform the experiments to test and extend those crucial findings in multiple mammalian species. The rat strain that we used is the Long Evans rats which are known to exhibit most robust learning performances in all laboratory rodent species and they are the favorite strain for many neuroscientists who conduct *in vivo* recording experiments. Also, successful making of transgenic rats for the neuroscience studies should also further enhance the experimental value of the rat model system from which a vast amount of neuropharmacological and behavioral data have been accumulated historically and facilitate the multi-level data comparisons [25], [26], [27], [28]. In addition, the large size of the rats (200–500 grams of body weight in comparison to the typical 20–30 grams of body eight for mice) also provides certain advantages in terms of large-scale *in vivo* neural recordings by using the existing electrode microdrive and head stage designs.

Our analyses reveal that the NR2B transgenic rats indeed exhibit superior memory function in comparison to the wild-type littermates. Conceivably, our demonstration of genetic enhancement in both mice and rats via NR2B overexpression greatly strengthens the notion that the NR2B gene is a valid drug target for improving memory function in both normal brains and patients with Alzheimer' disease or mild cognitive impairment [21], [29]. In the literature, some researchers suggest that optimal concentration of brain magnesium, which contributes to the voltage-gated opening of the NMDA channel, may increase the expression of NR2B subunit in cultured hippocampal neurons and lead to the juvenile form of the receptor [30], which may be one of the primary mechanisms for improved cognition in aged animals or brain injury models [31]–[34]. The up-regulation of NR2B by increased magnesium concentration seems to reflect a previous unrecognized mechanism by which the neurons deals with background calcium noise and opt for the efficient transmission of the NMDA receptor signals [30]. Thus, from this perspective, the optimal brain magnesium level should be systematically explored as a dietary means for its possible effects on improving memory via up-regulation of NR2B. This can be especially interesting in consideration of the reported magnesium deficiency in certain population including old people [35]. However, magnesium loading via diet should be carefully monitored to avoid side effects.

While the NMDA receptor is the primary receptor in gating the memory processes, other neurotransmitter systems can be also important for the regulation and modulation of various aspects of memory function, including motivation, attention, emotion, etc [36]–[38]. For example, the enhanced working memory in the transgenic rats raises an interesting question as what the role of dopamine in the prefrontal cortex might be. It would be also useful to investigate how those aspects are altered in the transgenic rats by carrying out various types of memory tasks requiring stronger attention, emotion, and motivation. Also in future experiments, it will be necessary to further dissect out various engaged neural circuits for memory enhancement [39]–[42].

In conclusion, our production and analysis of NR2B transgenic rats shows that the NR2B subunit is indeed a graded switch for the control of LTP but not for LTD in the CA1 region of the rat hippocampus. Moreover, the superior memory function in NR2B transgenic rats, together with the previously reported memory enhancement effects in NR2B transgenic mice [7], [20], [21], provides clear and consistent evidence that the NR2B subunit represents a rate-limiting genetic factor in gating NMDA receptor's optimal coincidence-detection and memory formation in the adult brains of multiple mammalian species. Genetic up-regulation of NR2B expression in the adult cortex and hippocampus is an effective means for rejuvenating synaptic plasticity and improving learning and memory.

## Materials and Methods

### Production and Biochemical Characterization of Transgenic Rats

The transgenic founders were produced by pronuclear injection of the linearized DNA into zygotes collected from Long Evans female rats. The genotypes of all offspring were analyzed by preparing tail DNAs, initially using southern blots and subsequently using PCR. The 5′ and 3′ primers for detecting NR2B transgene SV40 poly(A) sequence (505 bp) were 5′-AGAGGATCTTTGTGAAGGAAC-3′ and 5′-AAGTAAAACCTCTACAAATG-3′, respectively. Rat tail DNAs (about 1 µg) were amplified 30 cycles (1 min, 94°C; 45 sec, 55°C; 1 min, 72°C) on a thermal cycler. For detecting transgene mRNA, a SV40 poly(A) tail fragment was used for Northern blot. For Western blot, the antibodies against NR1, NR2A, and NR2B were purchased from Upstate Biotechnology. Synaptic membrane proteins were prepared from the rat forebrain. The samples were resolved on 7.5% SDS-polyacrylamide gels followed by immunoblotting with the above antibodies respectively, detected by peroxidase-labeled secondary antibodies and the ECL detection system (NEN Life Science products).

For *in situ* hybridization, rat brains were dissected and rapidly frozen. Crytostat sections (20 µm) were prepared and postfixed for 5 min in 4% PFA in PBS buffer (pH 7.5). The slices were hybridized to the [^35^S] oligonucleotide probe (5′-GCAGGATCCGCTTGGGCTGCAGTTGG ACCT-3′), which hybridizes to sequences present in the 5′ untranslated artificial intron region unique to the transgene.

### Electrophysiological Recordings on Hippocampal Slice Preparation

Rats (2–3 months old) were anaesthetized with sodium pentobarbital and sacrificed by decapitation. Transverse slices of the hippocampus (400 µm ) were cut by a tissue chopper and transferred to an chamber with artificial cerebrospinal fluid (ACSF) consisting of the following composition (in mM): NaCl, 120; CaCl_2_, 2; MgSO_4_, 25; NaHCO_3_, 1.0; Na_2_HPO_4_, 1.0; Glucose, 10, saturated with 95% O_2_ and 5% CO_2_. A bipolar tungsten stimulating electrode was place in the stratum radiatum to activate the Schaffer-collateral pathway projecting to CA1. A glass microelectrode (3–12 MΩ, filled with ACSF) was positioned also in the stratum radiatum to record presynaptic fiber volley and followed extracellular field potentials (fEPSP). Test responses were elicited at 0.03 Hz. After obtaining a stable baseline response for at least 15 min, LTP was induced by applying high frequency stimulation (100 Hz stimulation for 1 s). For inducing LTD, the standard 1 Hz protocol was used. Data were presented as the mean±SEM. Student's t-test was used for the statistical analysis.

### Novel Object Recognition Test

The protocol is similar to that published previously [7]. Rats were individually handled for 3 days and then habituated to an open-field box (40*40*20 cm) for 3 days. The object recognition task consisted of training and retention sessions. During the training session, two objects were placed equidistant from the center of the box and each rat was given 5 min to explore the box. The amount of time spent exploring each object was recorded. The rat was then returned to its home cage. During retention tests (retention for 1-hour, 1-day, 3-day, and 7-days), the trained rats were again placed individually in the box, in which one of the familiar objects was replaced by a novel object, and the rat was given 5 min to explore. The ratio of the amount of time spent exploring the novel object over the total time spent exploring both objects (preference index) was calculated to evaluate the recognition memory for each rat.

### Hidden-platform Water Maze

The protocol is as described previously [7]. Briefly, a circular pool (1.5 m in diameter) was filled with opaque liquid (constant temperature 25°C) made by addition of white powder. The hidden platform was 2 cm below the surface of the liquid and placed in the middle of one quadrant. The navigation of the rat, the escape latency and swimming length to the hidden platform were automatically recorded by Track Video Analysis System (Coulbourn instrument, USA). Three days before training, rats were handled and habituated to swim in the pool 60 seconds per day without the hidden platform. One training session (four trials) was conducted daily. During the training sessions, rats were required to locate the hidden platform by using visual cues surrounding the maze. The time taken to find the platform is measured. The experimenter would guide the rat to the platform if it failed during 60 seconds. Two transfer tests were carried out at the end of the third and fifth session respectively, in which the platform was removed and rats were allowed to swim for 60 seconds. The time spent in each quadrant was recorded. The behavioral performances were analyzed by Student's *t*-test.

### T-maze Spatial Working Memory Test

The protocol is as described previously [22]. The T-maze was made of black plexiglass and composed of a start arm (length 70 cm, width 20 cm and height 20 cm) with a start box (the first 20 cm of the start arm) and two identical goal arms (length 70 cm, width 20 cm and height 20 cm) with a food cup located 5 cm from the end of each goal arm. Before the training session, rats were housed individually with freely accessible drinking water, but maintained on a restricted food-pellet feeding schedule at approximately 85% of their pre-experimental body weight. They were habituated to the maze environment and were accustomed to reward foods (a small sugar pellet). Each trial consisted of a sample-run and a choice-run. On the sample-run, the rat was forced to enter either the left or right arm to get food (a small sugar pellet) while the other arm was blocked by a door. The direction of the forced run was random but no more than 2 times allowed in the same direction consecutively. On the choice-run, the blocked door was removed and the rat was allowed to choose either arm freely. The time interval between the sample- and the choice- run was 15 seconds during training. If rats enter the previously unvisited arm, the rats were rewarded before being placed back in the cage. Between each run the arms were quickly cleaned with 75% alcohol to remove the effect of olfactory. The training session lasted until the correct performance was stabilized at 85%. The delayed alternation was systematically prolonged to 1, 3, and 5 minutes. Four trials were conducted each day for two consecutive days. Behavioral performance was analyzed by a one-way ANOVA.

## Supporting Information

Figure S1 A NR2B Transgenic rat is shown, next to a NR2B transgenic mouse. The transgenic rats grow normally. The rats on Long Evans strain are ten to twenty times bigger than mice in body weight. Because of the large body size, the transgenic rats can be useful for conducting large-scale in vivo neural ensemble recordings during the freely behaving state.
(4.86 MB TIF)

Figure S2 The transgenic NR2B rats and wild-type littermates showed the comparable performances at the end of 5th training session. There is no longer any difference in place preference under either full cue or partial cue conditions.
(0.72 MB TIF)
