# Supplementary figures and images for: Genetic Enhancement of Memory and Long-Term Potentiation but Not CA1 Long-Term Depression in NR2B Transgenic Rats
Source: PLoS One. 2009 Oct 19;4(10):e7486. doi: 10.1371/journal.pone.0007486 (PMC2759522; doi:10.1371/journal.pone.0007486)

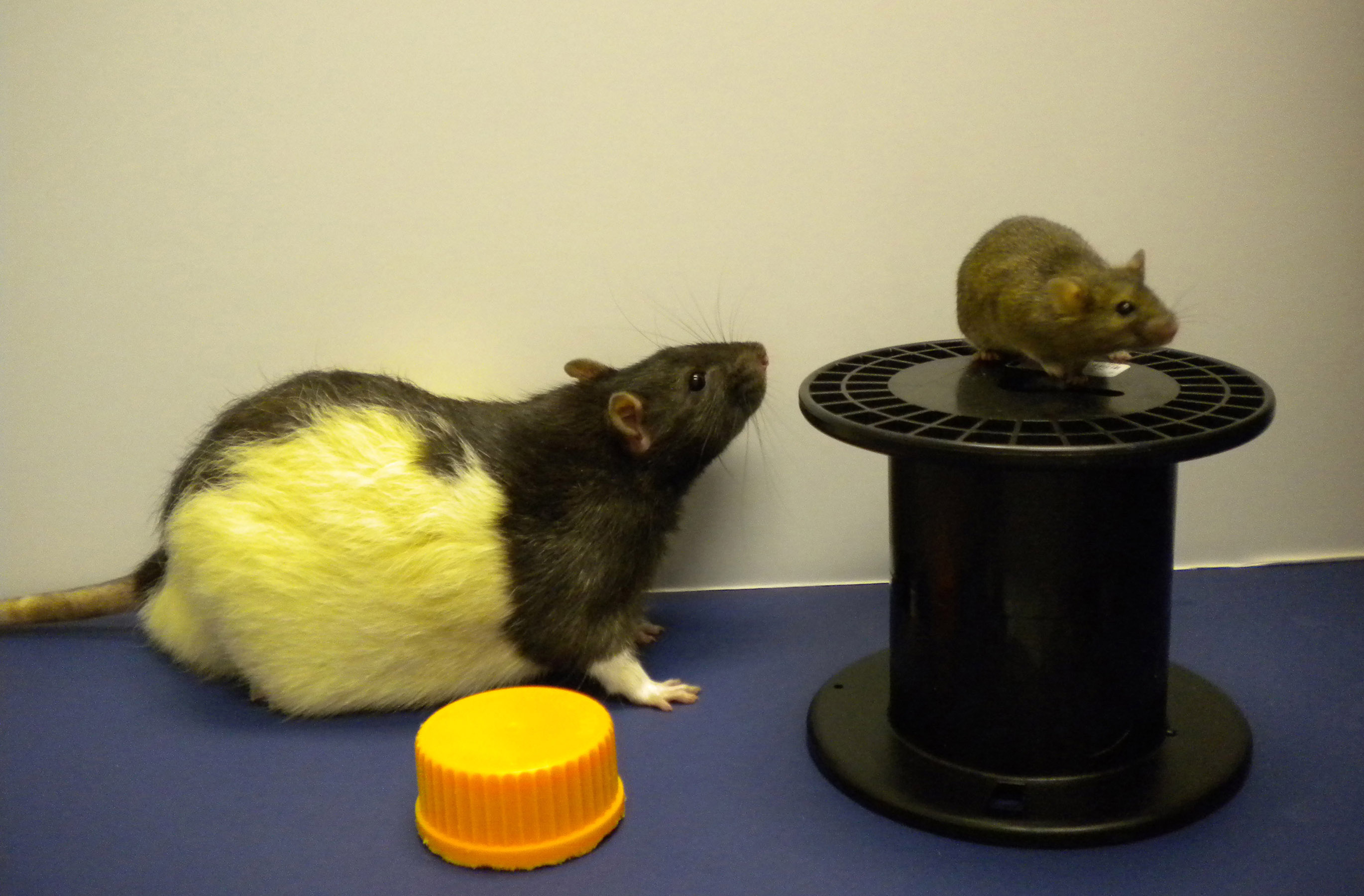

Supplement: Figure S1 — A NR2B Transgenic rat is shown, next to a NR2B transgenic mouse. The transgenic rats grow normally. The rats on Long Evans strain are ten to twenty times bigger than mice in body weight. Because of the large body size, the transgenic rats can be useful for conducting large-scale in vivo neural ensemble recordings during the freely behaving state. (4.86 MB TIF) [file pone.0007486.s001.tif]

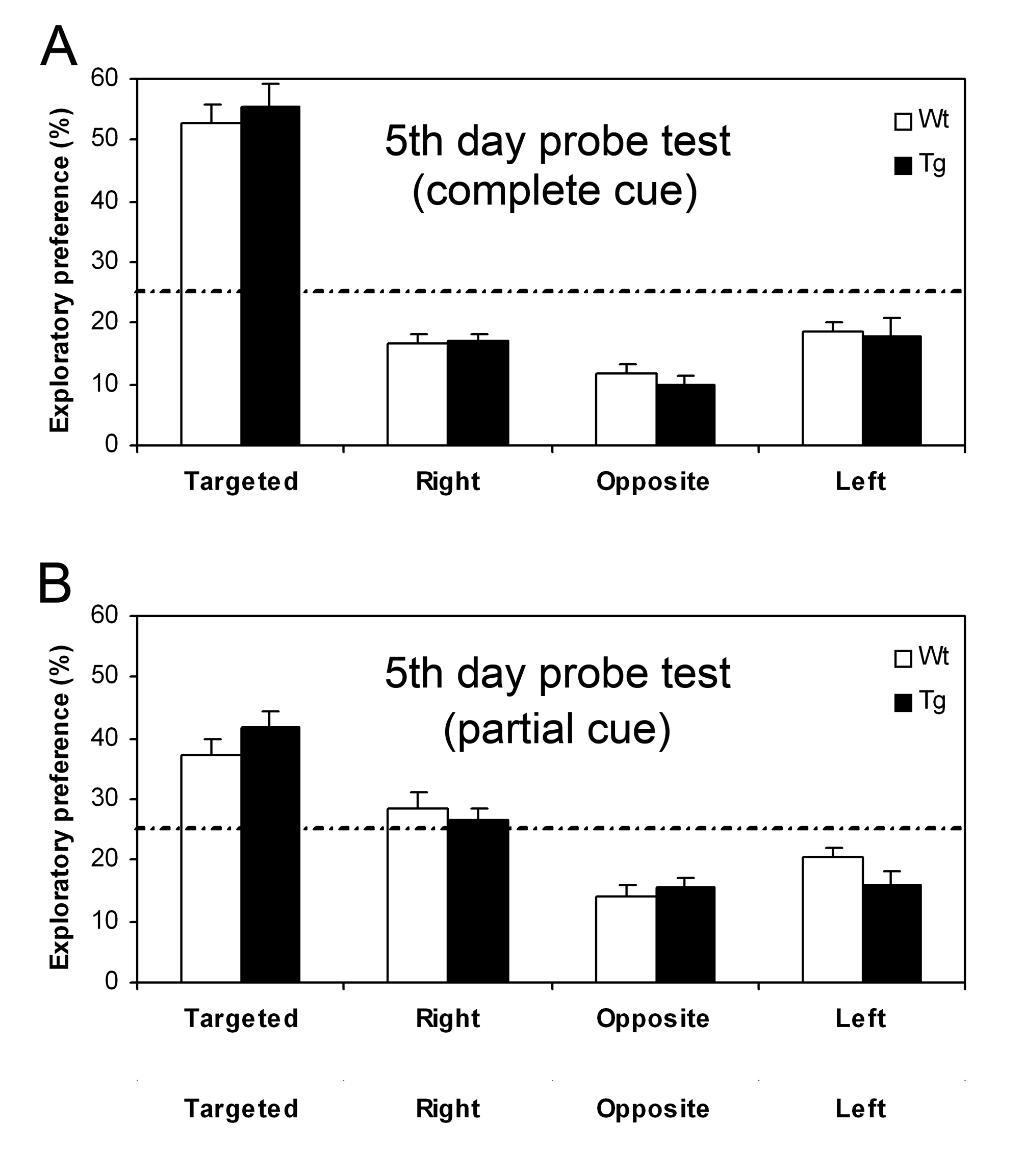

Supplement: Figure S2 — The transgenic NR2B rats and wild-type littermates showed the comparable performances at the end of 5th training session. There is no longer any difference in place preference under either full cue or partial cue conditions. (0.72 MB TIF) [file pone.0007486.s002.tif]
